# Gastric outlet obstruction in a patient with Bouveret’s syndrome: a case report

**DOI:** 10.1186/1756-0500-6-195

**Published:** 2013-05-12

**Authors:** Celso Nabais, Raquel Salústio, Inês Morujão, Francisco V Sousa, Eusébio Porto, Carlos Cardoso, Caldeira Fradique

**Affiliations:** 1 Department of Surgery, Hospital de São José, Centro Hospitalar de Lisboa Central, Serviço de Cirurgia 1, Rua José António Serrano, 1150-199, Lisboa, Portugal

**Keywords:** Bouveret’s syndrome, Gallstone ileus, Gastric outlet obstruction, Cholecystoduodenal fistula

## Abstract

**Background:**

Gallstone ileus accounts for 1% to 4% of cases of mechanical bowel obstruction, but may be responsible for up to 25% of cases in older age groups. In non-iatrogenic cases, gallstone migration occurs after formation of a biliary-enteric fistula. In fewer than 10% of patients with gallstone ileus, the impacted gallstones are located in the pylorus or duodenum, resulting in gastric outlet obstruction, known as Bouveret’s syndrome.

**Case presentation:**

We report an 86-year-old female who was admitted to hospital with a 10-day history of persistent vomiting and prostration. She was in hypovolemic shock at the time of arrival in the emergency department. Investigations revealed a gallstone in the duodenal bulb and a cholecystoduodenal fistula. She underwent surgical gastrolithotomy. Unfortunately, she died of aspiration pneumonia on the fourth postoperative day.

**Conclusion:**

This case shows the importance of considering Bouveret’s syndrome in the differential diagnosis of gastric outlet obstruction, especially in the elderly, even in patients with no previous history of gallbladder disease.

## Background

Gallstone ileus is a rare complication of gallstone disease, causing 1% to 4% of cases of mechanical bowel obstruction. In patients older than 65 years, this condition may cause up to 25% of cases of small bowel obstruction [1-3]. Gallstone ileus is 3 to 16 times more frequent in females than in males and has a high mortality rate (15% to 18%) because the patients are typically elderly with multiple comorbidities [1]. The most common location of gallstone impaction is the terminal ileum (50% to 75% of cases), with less than 10% impacting in the pylorus or duodenum [4]. Impaction in the pylorus or duodenum causes gastric outlet obstruction (GOO) with epigastric pain and postprandial vomiting. In 1896, Léon Bouveret described GOO caused by gallstone impaction in the duodenum or pylorus, which is now known as Bouveret’s syndrome. This is a rare clinical condition, with few cases reported in the literature [3]. Despite its rarity, it is important to consider this condition in a patient with GOO. We present here a case of Bouveret’s syndrome in which other comorbidities added to the diagnostic challenge.

## Case presentation

An 86-year-old Caucasian woman with previous history of coronary disease was admitted to the emergency department with a 10-day history of prostration, general malaise, anorexia, and persistent vomiting. She was hemodynamically unstable with a blood pressure of 53/42 mmHg, heart rate of 120 beats/min, respiratory rate of 23 breaths/min, and no fever. Crystalloid fluid resuscitation was effective in achieving hemodynamic stability. Physical examination showed that she was confused and disoriented, with clinical signs of poor peripheral perfusion and dehydration. Her abdomen was distended, with epigastric pain and tenderness, but no guarding or rebound. She had a reducible umbilical hernia with no signs of strangulation (2 cm defect). Breast examination revealed nipple inversion and a 7 cm diameter hard mass in the left breast which was fixed to the underlying tissues, and associated left axillary adenopathy.

She was diagnosed with GOO. At this time, we considered that her GOO might be due to malignancy such as primary gastric cancer or secondary breast cancer, or a benign condition such as peptic ulcer disease.

Laboratory testing showed normocytic normochromic anemia with hemoglobin 10.5 × 10 g/L (10.5 g/dL), leukocytosis with white cell count 11.2 × 10^9^/L (95.2% neutrophils), renal impairment with creatinine 176.79 μmol/L (2.0 mg/dL) and blood urea nitrogen 55.69 mmol/L (156 mg/dL), C-reactive protein 49.2 mg/L, sodium 149 mmol/L, potassium 3.4 mmol/L, and chloride 113 mmol/L. Her other results were unremarkable.

**Figure 1:**
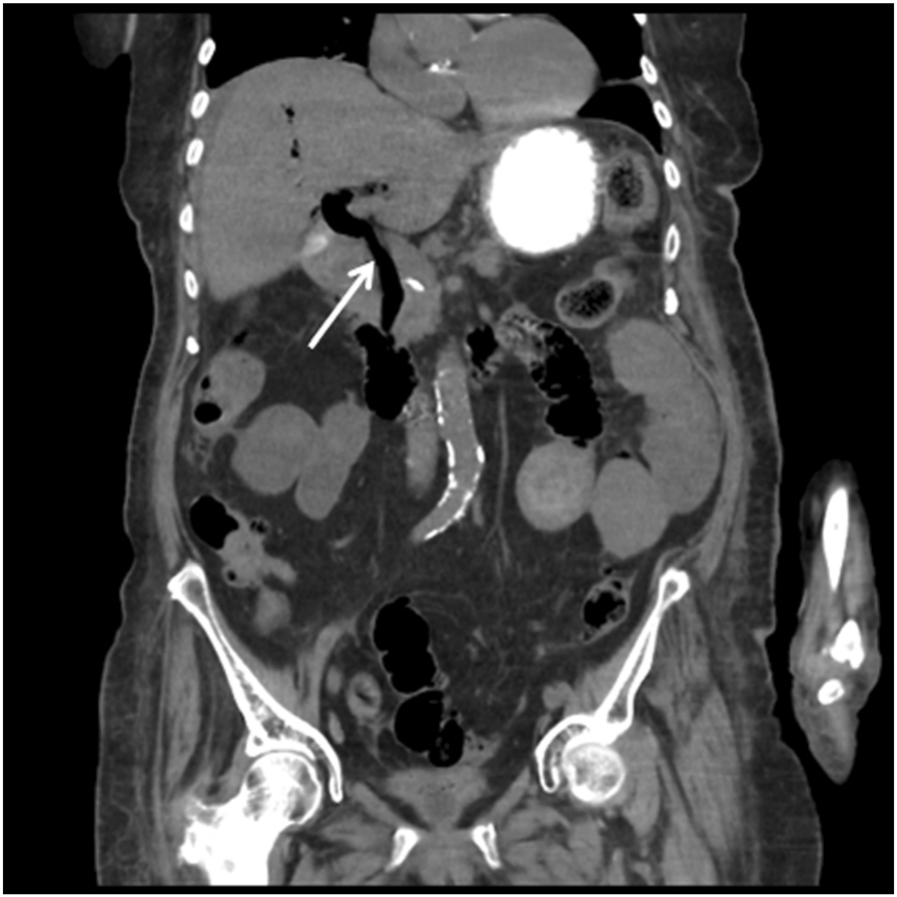
Computed tomography image (coronal view) showing a cholecystoduodenal fistula (arrow).

**Figure 2:**
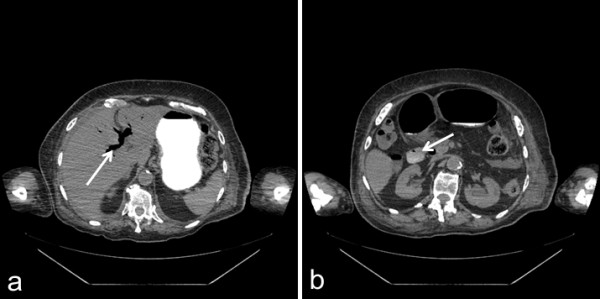
**Computed tomography image (axial view) showing Rigler’s triad. a** Gastric distension and pneumobilia (arrow). **b** Gallstone in the duodenal bulb (arrow).

**Figure 3:**
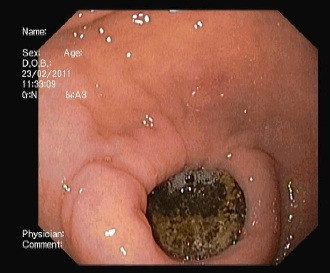
Esophagogastroduodenoscopy image showing a gallstone impacted in the duodenal bulb.

Plain abdominal X-ray did not show any significant changes. Abdominal ultrasonography revealed possible pneumobilia, but the gallbladder was not identified and there was no dilation of the common bile duct. Abdominal computed tomography (CT) with oral contrast showed pneumobilia, a cholecystoduodenal fistula associated with gallstone impaction in the duodenal bulb, and gastric distension (Figures 1 and 2). CT also revealed increased density of the mesenteric fat adjacent to the descending colon. No intra-abdominal free fluid, extraluminal air, colonic wall thickening, or fluid collections were observed. Esophagogastroduodenoscopy was performed as a minimally invasive therapeutic approach, but attempted endoscopic extraction of the gallstone using a basket and mechanical lithotripsy was unsuccessful because the large gallstone caused total obstruction (Figure 3).

**Figure 4:**
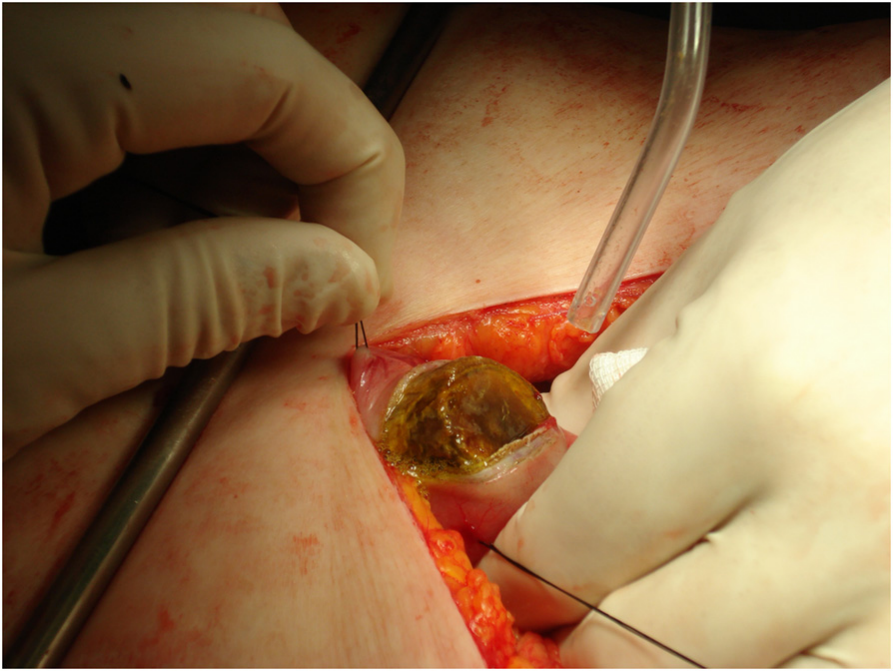
Gastrolithotomy.

**Figure 5:**
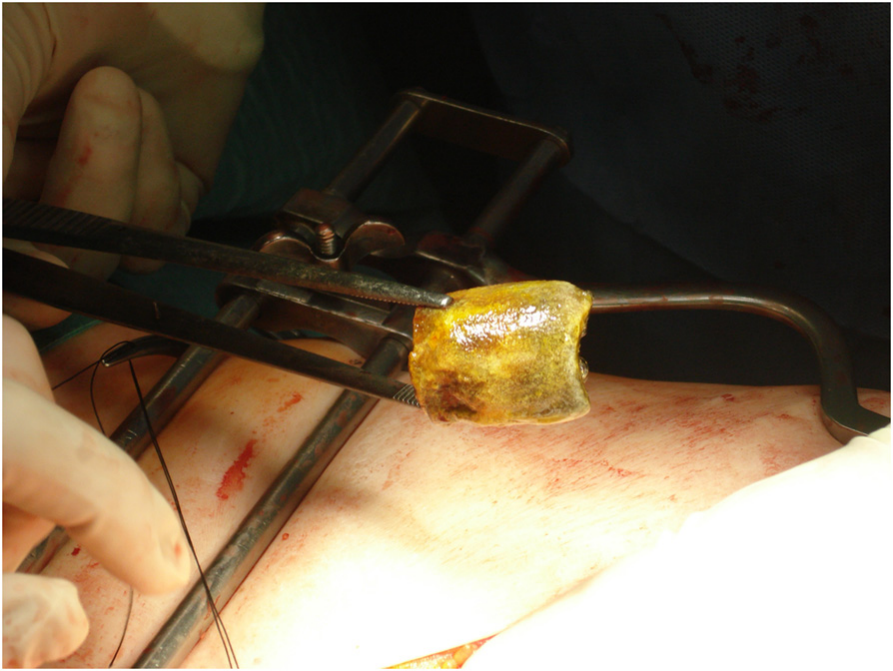
The extracted gallstone.

When the patient was stabilized at 6 days after admission, she underwent surgery. At laparotomy, a 2.5 × 3.5 cm cholesterol gallstone was found in the duodenal bulb. No other gallstones were found. There was no previous history of gallstone disease. Unexpectedly, there was also an inflammatory mass involving the sigmoid colon with abscess formation and purulent peritonitis, compatible with stage III (Hinchey classification) acute diverticulits [5]. We performed anterior gastrotomy in the antrum, extracted the gallstone by milking, and closed the gastrotomy using a double row of sutures (Figures 4 and 5). We then performed a Hartmann’s procedure.

The patient was stable after surgery, but she removed her nasogastric tube on the first postoperative day, with subsequent vomiting and aspiration. She developed aspiration pneumonia. Her clinical condition worsened, and she died of aspiration pneumonia on the fourth postoperative day.

Histopathological examination of the resected colon specimen confirmed diverticulitis. Histopathological examination of a left breast core biopsy specimen revealed invasive ductal carcinoma.

## Conclusion

Bouveret’s syndrome is a specific form of gallstone ileus, caused by gallstone impaction in the pylorus or duodenum resulting in GOO. This rare disorder (3% to 5% of cases of gallstone ileus) is usually associated with a cholecystoduodenal fistula, which allows the passage of large a stone to the digestive tract [6]. The absence of a preceding history of gallstone disease does not exclude this entity, as such a history was present in only 27% of patients in some series [2]. Clinically, it presents as GOO, with predominant symptoms of nausea, postprandial vomiting, and epigastric pain. Inclusion of this disorder in the differential diagnosis of GOO (together with malignancy and peptic ulcer disease, which are the main causes of GOO) is challenging due to its rarity and vague, nonspecific signs and symptoms.

Classically, Rigler’s triad (signs of intestinal obstruction, ectopic gallstone, and pneumobilia) on plain abdominal X-ray is used to diagnose gallstone ileus [7]. However, this triad is present on plain X-rays in less than 15% of cases, and CT scan is therefore the most widely used imaging technique, allowing visualization of Rigler’s triad in about 78% of cases [8]. Recently, magnetic resonance cholangiopancreatography has been used increasingly to diagnose this condition. Esophagogastroduodenoscopy may be performed for both diagnostic and therapeutic purposes [9]. However, the ability to remove impacted gallstones endoscopically is still limited [2].

Surgery is needed in more than 90% of patients with Bouveret’s syndrome, and is therefore the mainstay of treatment. There are two main surgical approaches. In patients with significant comorbidities or who are in a critical condition, a two-stage procedure is usually performed, consisting of urgent enterolithotomy or gastrolithotomy to resolve the obstruction, followed by cholecystectomy and fistula closure at a later date. This approach carries a 5% risk of recurrence of gallstone ileus before the second stage. Other possible complications such as cholecystitis, cholangitis, gallbladder carcinoma or hemorrhage can occur [10,11]. The other approach is a one-stage procedure, which combines enterolithotomy, cholecystectomy, and fistula closure. This surgery is more invasive and carries a higher risk, and is usually only performed in selected low-risk patients [10,12]. Laparoscopic surgery by an experienced surgeon is also possible in selected patients [2].

Recently, nonsurgical treatments have had an increasing role in patients at high risk for perioperative complications. Such treatments include endoscopic extraction of the gallstone with or without mechanical lithotripsy, and extracorporeal shock wave lithotripsy. However, these treatments are technically difficult, and patients may still require surgery [2,11]. In our patient, attempted extraction was not successful.

Bouveret’s syndrome still has a high mortality rate (up to 35% in some series), attributed mainly to the characteristics of the affected population who tend to be elderly with multiple comorbidities, and delayed diagnosis. Surgical treatment also carries significant risks, with mortality rates of about 17% for the one-stage procedure and 12% for enterolithotomy alone [10,12].

This case shows a rare complication of cholelithiasis, which is a common disease. Bouveret’s syndrome is an unusual and clinically distinct form of gallstone ileus, which results in GOO with vague and nonspecific symptoms. The diagnosis of this condition is therefore challenging and is usually delayed, contributing to the high mortality rate in the typically elderly patients with multiple comorbidities. Even after surgical treatment, the mortality rate remains high due to the baseline patient characteristics. In this case, the patient had both GOO and acute diverticulitis. Her diverticulitis, which was diagnosed during surgery, contributed to her poor outcome. The absence of a clinical history suggestive of diverticulitis (no lower abdominal pain and no change in bowel habit), presence of radiological findings indicating Bouveret’s syndrome, and no radiological findings of acute diverticulitis (probably because of the early phase of the disease) contributed to the delayed diagnosis.

This case highlights the importance of considering Bouveret’s syndrome in the differential diagnosis of GOO, especially in the elderly population. Even if uncommon, the presence of two distinct intra-abdominal pathologies is possible and can become a diagnostic challenge.

## Consent

Written informed consent was obtained from the patient’s next of kin for publication of this case report and accompanying images. A copy of the written consent is available for review by the Editor-in-Chief of this journal.

## Abbreviations

CT: Computed tomography; GOO: Gastric outlet obstruction.

## Competing interests

The authors declare that they have no competing interests.

## Authors’ contributions

RS, IM, FS, EP, CC, and CF analyzed and interpreted the patient data, and contributed to the design and revisions of the manuscript. CN analyzed and interpreted the patient data, wrote the manuscript, and obtained informed consent. All authors read and approved the final manuscript.
